# Ultrafast snapshots of terahertz electric potentials across ring-shaped quantum barriers

**DOI:** 10.1515/nanoph-2023-0538

**Published:** 2023-11-02

**Authors:** Taehee Kang, Richard H. J. Kim, Jinwoo Lee, Minah Seo, Dai-Sik Kim

**Affiliations:** Sensor System Research Center, Korea Institute of Science and Technology, Seoul, 02792, Republic of Korea; Ames National Laboratory, Ames, IA 50011, USA; KU-KIST Graduate School of Converging Science and Technology, Korea University, Seoul, 02841, Republic of Korea; Department of Physics and Astronomy, Seoul National University, Seoul, 08826, Republic of Korea; Department of Physics, Long-Wavelength Nanotechnology Laboratory, and Quantum Photonics Institute, Ulsan National Institute of Science and Technology (UNIST), Ulsan 44919, Republic of Korea

**Keywords:** light-field–driven electron tunneling, metal–insulator–metal structures, nano resonators, terahertz electric potential mapping, terahertz imaging, ultrafast imaging

## Abstract

Probing the time evolution of the terahertz electric field within subwavelength dimensions plays a crucial role in observing the nanoscale lightwave interactions with fundamental excitations in condensed-matter systems and in artificial structures, such as metamaterials. Here, we propose a novel probing method for measuring terahertz electric potentials across nanogaps using a combination of optical and terahertz pulse excitations. To achieve this, we employ ring-shaped nanogaps that enclose a metallic island, allowing us to capture tunneling charges when subjected to terahertz electromagnetic pulse illumination. By controlling and manipulating the terahertz tunneling charges through a focused optical gate pulse, we can obtain the terahertz potential strength as a function of spatial coordinates and time delays between pulses. To accurately quantify the time evolution of terahertz electric potential across quantum barriers, we carefully calibrate the recorded nonlinear tunneling current. Its on-resonance and off-resonance behaviors are also discussed, providing valuable insights into the antenna’s characteristics and performance.

## 1 Introduction

The terahertz (THz) electromagnetic wave holds immense promise for a wide range of applications, from advanced communication systems such as 6G technology [1, 2] to biomedical sensing [3–5] and imaging [6–10], and security screening techniques [11, 12]. With harnessing the full potential of THz radiation, it requires a deep understanding of its interactions with matter, especially at the nanoscale. Conventional imaging methods have been hindered by the diffraction limit due to its long wavelength characteristics, preventing the precise visualization and study of THz waves at subwavelength dimensions. To overcome this limitation and induce efficient THz light–matter interactions on the nanoscale, researchers have been exploring innovative near-field imaging techniques including tip-based scanning [13–17], electro-optic sampling [18], and photoemission microscopy techniques [19, 20]. The versatility of these methods allows us to explore a wide range of condensed matter systems, with artificial structures such as metamaterials [21–25], and even quantum barriers [26]. By capturing the ultrafast variations of THz electric potentials, these imaging techniques pave the way for a deeper understanding of low-energy elementary excitations in materials and the efficiency of their coupling with nanostructures [27–29].

Where the THz electromagnetic waves irradiate a nanoscale metal structure, THz near field is greatly enhanced in a confined space, extending far beyond the traditional diffraction limit. This near field not only enables THz nanoscopy of structures extremely smaller than the wavelength of light, transcending the limitations of diffraction but also provides an exceptional platform for studying strong-field–driven electro-optic dynamics. Recent advancements in controlling the carrier-envelope phase of light have further expanded the possibilities. Specifically, the fusion of THz technology with cutting-edge microscopy and nanoscale control has led to the observation of atomic-scale ultrafast dynamics as well [13, 14, 30–32]. Additionally, by irradiating tunnel junctions with THz pulses, researchers have achieved ultrafast tunneling currents, which can be coherently manipulated by THz modulations [27, 29].

In our previous study (Ref. [33]), we have demonstrated a method to control and probe ultrafast tunneling currents with closed loops of tunneling barriers and enabled full-wave rectification of incoming THz pulses. Due to the nonlinearity of tunneling process, finding direct connection between measured tunneling current and applied THz potential across the barrier using optical gating pulses is not straightforward with conventional electronic apparatus. In this work, we propose a method to experimentally quantify the absolute THz near-field potential by converting the measured ultrafast tunneling currents from ring-shaped nanoantennas using femtosecond gating pulses. To ensure accuracy, we carefully measure and calibrate the actual THz potential applied across the barriers of the loop. This calibration is achieved through precise measurements of THz tunneling currents and time-resolved optical gating, enabling us to quantitatively analyze the THz electric potential with exceptional precision and reliability. Our investigation mainly demonstrates spatiotemporal mapping of THz near fields, with which sub-picosecond and micrometer resolution probing reveals resonant and nonresonant nanoantenna field patterns. Our findings have the potential to impact diverse fields, from nano-electronics and quantum devices to ultrafast photonics and beyond, as we explore new frontiers in the manipulation and understanding of electro-optic phenomena at the nanoscale.

## 2 Results and discussions

**Figure 1:**
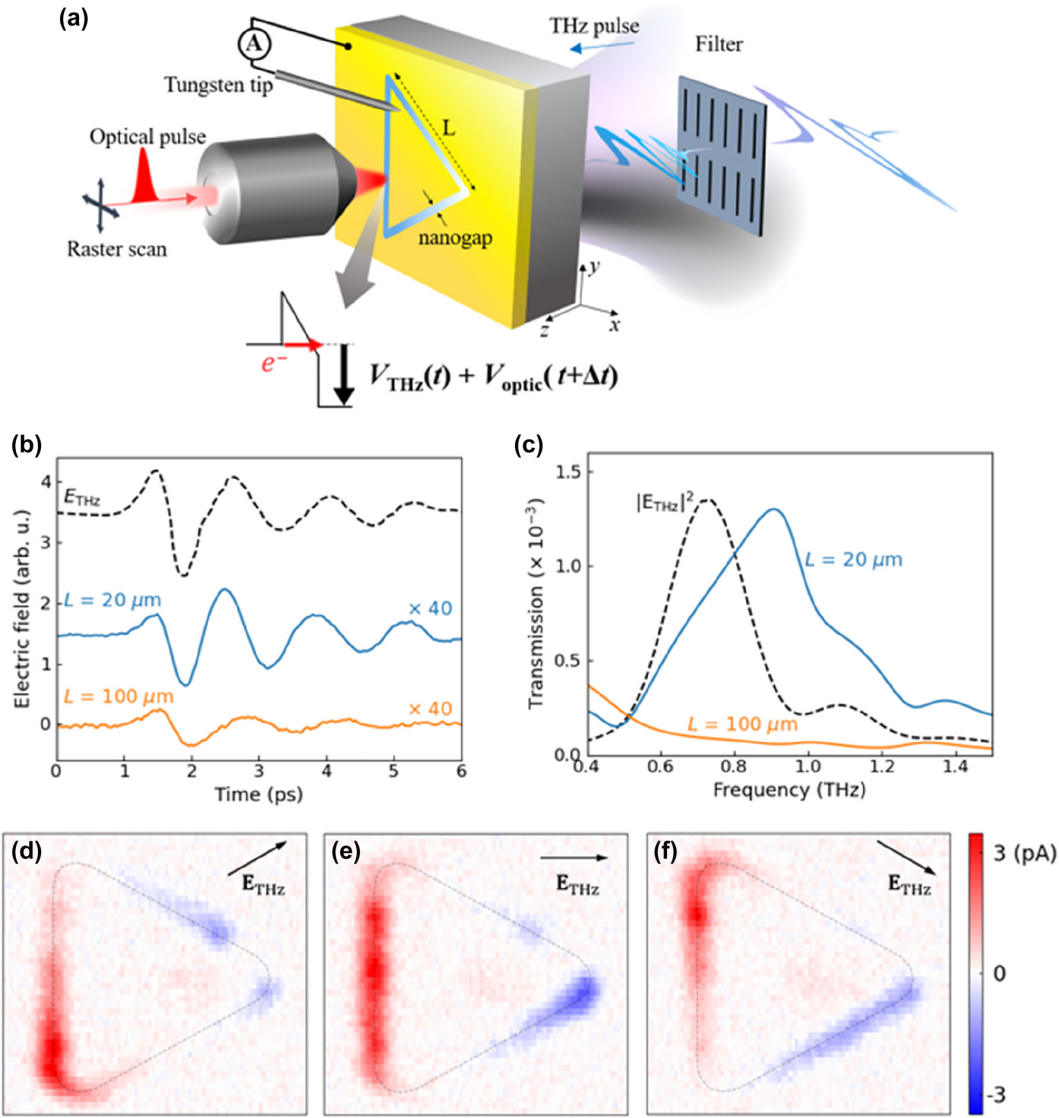
Experimental scheme for THz potential measurements for a loop-shaped nanoantenna. (a) Experimental scheme of THz electric potential mapping across ring-shaped tunneling barriers with an arm length of *L*. (b) Time trace of incident THz pulse (black dashed), transmitted time trace of a triangular loop of arm size, *L* = 20 μm (blue solid), and *L* = 100 μm (orange solid). Note the scaling factor of 40 for each loops to display the time traces clearly. (c) Transmission spectra for *L* = 20 μm and *L* = 100 μm loops normalized by the incident THz pulse spectrum. (d) Measured tunneling current maps of *L* = 20 μm loop for several incident THz polarization angles of + 30°, (e) 0°, and (f) −30° black dashed lines are guided to the eye indicating triangular-shaped barriers.

An experimental scheme of probing ultrafast electric potential on ring-shaped quantum barriers is shown in Figure 1(a). These barriers consist of a few nanometer-thick Al_2_O_3_ layers vertically embedded in a 100-nm-thick Au film, fabricated on a 500-μm-thick quartz substrate by atomic layer deposition technique [33, 34]. The barriers form a triangular-shaped loop encasing a metallic island [33]. The central metallic island is electrically isolated by the insulating surroundings, i.e., the Al_2_O_3_ layer covers the sides of the island, and quartz substrate and air the top and bottom planes. Then, the closed-loop geometry of the tunneling barriers provides the unique property of charge rectification under a single electromagnetic pulse excitation, depending upon the shape of the loop [33]. Under the intense THz pulse illumination, the presence of a nanometer-sized gap enhances the THz near field and triggers the quantum tunneling of free electrons in metals across the Al_2_O_3_ barriers. To generate the THz pulse, we utilized tilted-pulse-front optical rectification from LiNbO_3_ crystal derived from amplified Ti:sapphire laser pulses of 1 kHz repetition rate at center wavelength of 800 nm with 35 fs pulsewidth. THz polarizations and amplitudes are controlled by a pair of wire grid polarizers.

With band-pass filtered at 0.8 THz, a few-cycle THz pulse with a peak field strength of 50 kV/cm interacts with the samples. Narrowing the THz pulse bandwidth down to 0.25 THz provides several cycles of THz pulses in the time domain to clearly exhibit the oscillatory feature of the THz potential in the barriers. The time trace of the THz pulses was detected using the free-space electro-optic sampling method using 200-μm-thick (110) ZnTe crystal. Figure 1(b) shows measured electro-optic sampling time traces of incident THz pulse after the spectral filter and transmitted amplitudes of triangle samples. We designed arm length *L* of our samples to be *L* = 20 μm and *L* = 100 μm, respectively. The resonance of *L* = 20 μm sample is mostly overlap with the incident THz pulse, whereas the resonance of *L* = 100 μm sample is far below from the incident THz pulse (Figure 1(c)).

Following a single THz pulse interaction with the barriers, the cumulative tunneling charges along the entire perimeters of the loop are gathered inside the metallic island. These rectified charges carry information of time-integrated THz electric potential across the barriers driven by the THz pulse. To acquire dynamics of rectified charges, i.e., time-dependent THz potential across the barriers, we introduced a focused optical gating pulse at a specific loop position as a function of time delay Δ*t* relative to the THz pulse. The role of the optical pulse is to apply an additional electric potential across the barrier on a micrometer-sized spot. Compared to the tunneling currents driven by the THz pulse only, the introduction of the optical pulse gives additional currents. These optically driven tunneling charges respond depending on the strength of quasi-static THz potential, therefore, revealing information on the THz potential at specific times and positions across the barrier. Only the vertically polarized optical pulse interacts with the nanogaps [33]. Due to the closed-loop geometry of the barrier, the optical pulses were circularly polarized to interact with the loop in a position-independent manner.

Through raster-scanning the optical pulse, we recorded maps depicting the distribution of THz-driven tunneling current across a triangular-shaped barrier. As the incoming THz spectrum closely matches with the fundamental resonance mode of the loop, one anticipates a sinusoidal field distribution along the loop perimeter. The oscillatory features such as the peak and node of the near field profile are clearly seen depending on the incident polarization direction of 30° (Figure 1(d)), 0° (Figure 1(e)), and −30° (Figure 1(f)). One can also check the polarity of the potential, i.e., inward or outward from the central island, by observing the direction of the measured current (as differently colored in the maps). The current maps exhibit a monotonic trend of the measured THz potential, yet are not quantitatively evaluated due to the intrinsic nonlinearity of the tunneling process. To handle the nonlinear behaviors, we carefully calibrated the measured current under the THz and optical pulse excitation. We leveraged the comprehensive integral expression of the electron tunneling current density, ***J***, across metal–insulator–metal tunneling barriers from Simmons [35],

(1) $$\begin{array}{ll} J\left(V\right) & =\frac{4\pi me}{h^{3}}\left[\int_{0}^{\eta }D\left(E\right)\left(\eta -E\right)dE\right.\\ & \;\left.-\int_{0}^{\eta -eV}D\left(E\right)\left(\eta -eV-E\right)dE\right], \end{array}$$

where *V* = *V*_THz_ + *V*_optical_ is the total applied electric potential by THz and optical pulses, *m* is the electron mass, *e* is the elementary charge, *h* is the Plank constant, *η* is the Fermi level of the gold (5.53 eV), $D\left(E\right)=\exp\left(-4\pi \Delta w\sqrt{2m\left(\eta +\phi_{m}-E\right)}/h\right)$ is the tunneling probability factor, $\phi_{\text{m}}=\int_{w_{1}}^{w_{2}}dx\left(\phi \left(x\right)+\phi_{i}\left(x\right)\right)/\Delta w$ is the mean value of barrier height, $\phi \left(x\right)=\phi -eVx/w$ is the barrier potential profile affected by applied potential *V*, $\phi_{i}\left(x\right)=-1.15e^{2}w^{2}\ln2/16\pi \varepsilon wx\left(w-x\right)$ is the approximated form of barrier modification by image force, *w* is the thickness of the barrier, *ε* is the dielectric constant of the insulating layer, and Δ*w* = *w*_2_ − *w*_1_ is the effective barrier width of which *w*_1_ and *w*_2_ are zeroth of $\phi \left(x\right)+\phi_{i}\left(x\right)=0$ .

Once we identify the relationship between the measured currents and THz potential amplitudes, we can directly use the measured tunneling currents to map the THz potentials. To achieve this, we employed a detailed calibration procedure using the measured tunneling currents and equation (1). First, we extracted the barrier height of the Al_2_O_3_ layer used in our experiment (2.2 eV) and layer thickness (5 nm for *L* = 20 μm and 4 nm for *L* = 100 μm) from *I*–*V* data acquired from DC measurements (Keithley 2450) using equation (1). To quantitatively simulate the tunneling currents driven by the THz and optical pulses, it is crucial to determine the probing length scale along the loop perimeter. The dominant contribution of spatial current flow by the focused optical pulse comes from the central part of the Gaussian spot. Therefore, we accounted for the size of the effective probing spot as a function of applied potential strengths using equation (1). For example, the original optical spot FWHM of 3 μm resulted in the actual interaction size of 1.4 μm. Next, we simulate the optical tunneling currents driven by the optical pulse. The optical pulse transient was approximated as a transform-limited Gaussian pulse of 170 fs pulse width (measured independently). By calculating tunneling current as a function of time using equation (1) and integrating during the optical cycles, finally we calculate tunneling currents per each laser pulses, i.e., dividing the integrated rectified charges by 1 ms [33].

**Figure 2:**
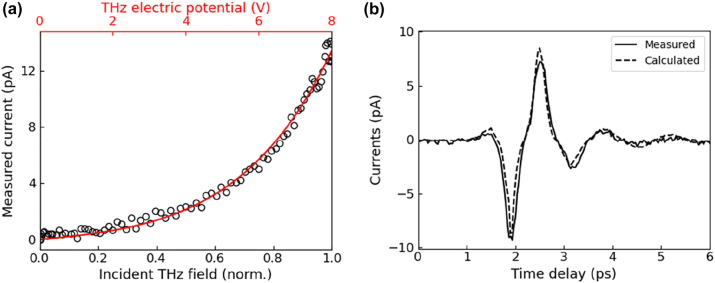
Calibrating THz potentials with measured tunneling currents. (a) Measured tunneling current of the *L* = 20 μm sample as a function of incident THz field strength (black circle) and calculated tunneling current with the optical potential of 16.4 V and maximum THz potential of 8 V across 5 nm barrier (red line). (b) Measured tunneling current time-trace for *L* = 20 μm sample (solid) under the THz and optical pulse illuminations by varying the time delay between two pulses and calculated tunneling current (dashed).

Figure 2(a) displays the measured tunneling current for the *L* = 20 μm sample as a function of incident THz field strength (black circles) and calculated curve describing relation between THz electric potential and measured currents (red line). Here, we illuminated the sample at specific point on the loop where THz potential reaches maximum with optical pulse energy of 3 nJ and measured tunneling currents by sweeping incoming THz field strength by varying the angle of wire-grid polarizers. From the curve-fitting process using equation (1) with free parameters of *V*_THz_ and *V*_optical_, we arrived at a unique curve as shown in red line (Figure 2(a)). The fitting curve directly connects the measured tunneling current with the THz electric potential across the gap for a given optical pulse energy and spot size. Therefore, we acquired the calibration curve by placing the optical pulse at a maximum THz potential position in the loop, before performing potential mapping measurements.

As a proof of concept, we present a measured tunneling current transient and compared with calculated current using the driving electric fields measured from electro-optic sampling signal of *L* = 20 μm in Figure 1(b). Solid line of Figure 2(b) shows the measured tunneling current transients acquired by varying the time delay between THz and the optical pulse. Here, the optical pulse was fixed at a specific position of the loop. Based on equation (1) with a peak THz potential of 6.8 V and optical potential of 14 V, the simulated tunneling current is shown in the dashed line in Figure 2(b), exhibiting good agreement with the experimental result. One can notice the effect of tunneling nonlinearity from the distorted oscillatory feature compared with the driving THz field transient shown in Figure 1(b) for the case of *L* = 20 μm.

**Figure 3:**
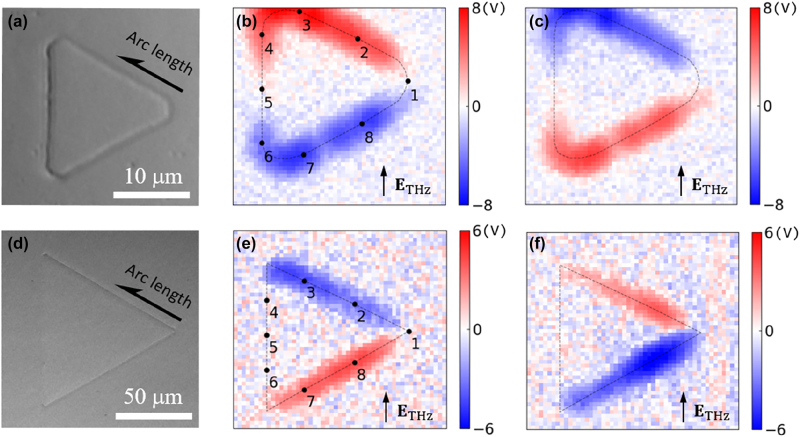
Ultrafast snapshots of THz electric potentials across quantum barriers along triagular-shaped loops. (a) Optical microscope image (oblique illumination) of the *L* = 20 μm sample. Measured THz electric potential maps at a time delay of (b) 0.3 ps and (c) 0.9 ps between THz and optical pulses. (d) Optical microscope image of *L* = 100 μm sample. Corresponding THz potential maps at a time delay of (e) 0.3 ps and (f) 0.9 ps of the *L* = 100 μm loop. Black dashed lines are a guide to the eye for the triangular loops. Several spatial probing points as numbered in (b) and (e) were chosen to display the time-dependent potential, which will be presented in Figure 4.

Utilizing the calibration curve, we present snapshots of the THz potential across the barriers along the loops. Figure 3(a) shows the optical microscope image of the *L* = 20 μm sample. For all measurements, the THz polarization was fixed at 90°, and the data sets were taken during a 1.8 ps time delays with a 100 fs step. The optical pulse energy illuminated to the sample was fixed to 3 nJ for *L* = 20 μm with spotsize of 3 μm, and 54 nJ for *L* = 100 μm sample with spotsize of 13.5 μm, respectively. Generally, we expect larger tunneling currents for higher optical field strength, thus lower the THz threshold potential for imaging. The relation is directly given by the tunneling equation (1) where the potential strength across the gap *V* is applied by both the *V*_THz_ and *V*_optical_. However, we fixed the optical pulse energy at moderate value (3 nJ for *L* = 20 μm sample with spotsize of 3 μm and 54 nJ for L = 100 μm sample with spotsize of 13.5 μm, respectively) to avoid any damages or thermal issues during the raster scan. The THz electric potential maps are displayed at time delays of 0.3 ps  (Figure 3(b)) and 0.9 ps  (Figure 3(c)), separated by a half cycle of THz pulse of 600 fs. The sign of the potential indicates the relative direction of the near field applied to the barrier corresponding to the central island, i.e., plus for the outward direction and minus for the inward direction from the island. Similarly, the THz electric potential maps were taken for *L* = 100 μm loop (optical image is in Figure 3(d)) and displayed at time delays of 0.3 ps  (Figure 3(e)) and 0.9 ps  (Figure 3(f)). Recorded maximum THz potential reaches 8 V for the *L* = 20 μm loop and 6 V for the *L* = 100 µm loop, respectively. The minimum THz potential strength we can measure was determined by the background noise in our current measurement. For instance, average fluctuation in potential map shown in Figure 3(b) is ∼0.5 V, which indicate minimum discernable potential value in the measured snapshots.

We double-checked the measured THz potential amplitudes with far-field electro-optic sampling signals. A coverage ratio of 6.33 × 10^−5^ of the *L* = 20 µm sample, together with a measured far-field transmitted amplitude of 0.018 in the time domain, gives an average peak potential of ∼7.9 V along the loops, estimated by Kirchhoff integral formalism [21, 36]. For the *L* = 100 µm loop, maximum potential reaches about 6 V in the maps, and the independent far-field analysis gives ∼6.4 V with sample coverage of 2.5 × 10^−5^ and transmitted amplitude of 0.008. Therefore, those far-field estimations match quite accurately with the measured THz potentials. Additionally, the curve-fitting procedure gives optical potential values of 16.4 V for the *L* = 20 µm sample, and this value corresponds to the optical field enhancement of 4.8 at 800 nm wavelength inside a 5 nm gap. This factor is well matched with the values from previous studies of optical field enhancement inside metallic nanogaps [37], thereby consistently validates the accuracy of our calibration method.

**Figure 4:**
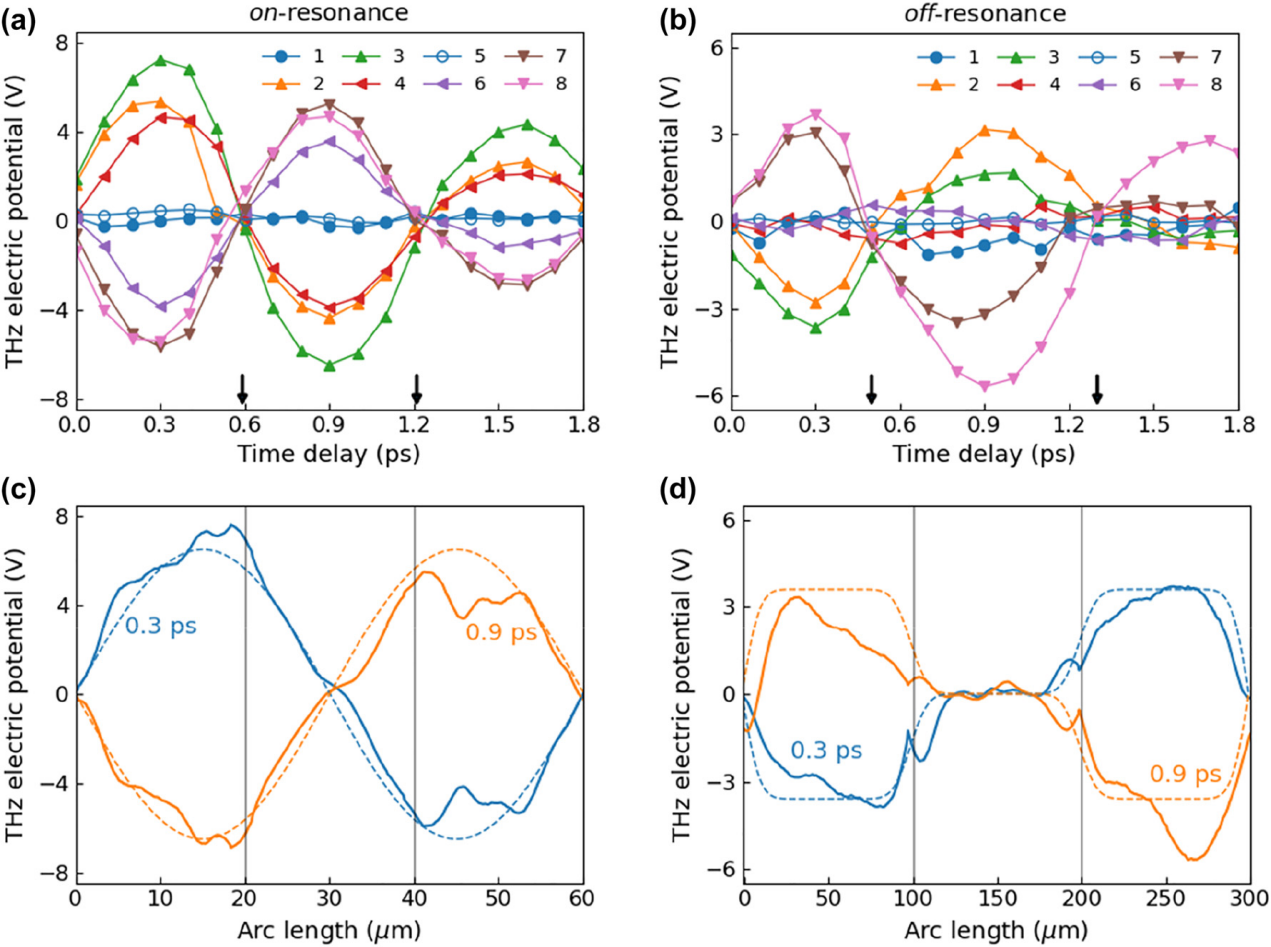
Potential dynamics for resonant/nonresonant nanostructures. (a) *L* = 20 µm and (b) *L* = 100 µm. Black arrows denote node positions of the oscillatory dynamics. Legend indicates the positions where the time-dependent potentials were measured. Electric potential profiles along the loop perimeter of (c) *L* = 20 µm and (d) *L* = 100 µm samples at specific time delays (0.3 and 0.9 ps). The dashed lines show the Gaussian-convoluted sine function (c) and square-wave–shaped function (d). Vertical black lines indicate the vertex positions of each triangle.

In addition to the absolute values of THz electric potential, the measured potential maps for each loop exhibit different waveguide mode patterns and dynamics. Figure 4(a) and (b) shows dynamics of THz potentials at specific positions in the loops, annotated by black dots in Figure 3(b) and (e). Especially, the position numbers, 1 and 5, indicate nodes of oscillation determined by the incident polarization. Therefore, these points exhibit mostly suppressed amplitudes during the excitation. The resonant loop of *L* = 20 μm exhibits well-defined driving pulse transient in time dynamics for all positions except the nodes (2–4 and 6 to 8 in Figure 4(a)) and sinusoidal oscillation along the loop (Figure 4(c)). The dashed lines in Figure 4(c) represent sine functions convoluted by Gaussian function with 3 μm half-width reflecting the probing pulse spot size. After a THz driving pulse half cycle (about 600 fs), the potential profile were reversed with each other. Also, the time delay between the nodes (marked by black arrows) is almost equal to the driving pulse half cycle. On the other hand, the *L* = 100 μm loop exhibits different overall behavior. Since the incident THz spectra is off-resonant with the *L* = 100 µm loop, the time dynamics of potentials show an elongated cycle period compared to the driving transient, as shown in the node positions of 0.5 and 1.3 ps in Figure 4(b). Each arm of the triangles behaves as independent slits with each other in contrast to the resonant case of *L* = 20 μm. The arm parallel to the incident polarization, i.e., arc length from 100 to 200 μm, was almost suppressed during THz excitation, as shown in Figure 4(d). The other arms of the loop show almost constant amplitude profiles, which are represented by Gaussian-convoluted (half-width of 13.5 μm) square-wave–like functional shape.

## 3 Conclusion

In conclusion, we probed THz electric potential across the quantum barriers by combining optical and terahertz pulse excitations. This method employs ring-shaped nanogaps facilitating the observation of rectified tunneling charges. The THz potential strength as a function of space and time delays was extracted from the manipulation of these charges. Across quantum barrier, we meticulously calibrate the nonlinear tunneling current to ensure the accurate THz electric potential evolution. On-resonance and off-resonance behaviors of THz potential across the barrier were also intensively discussed. The synergistic influence of the THz and optical pulses can offer a comprehensive view of the intricate dynamics of THz potential variations across the ring-shaped barrier, thereby enriching our understanding of the underlying phenomena. Overall, this innovative probing method offers a powerful tool for studying and visualizing THz electric potentials at the nanoscale, overcoming the diffraction limit. The ability to quantify the time evolution of THz electric potentials across quantum barriers and validate the concept through nanogap further demonstrates the potential applications of THz cameras and imaging tools.
